# Thermal Efficiency in Laser-Assisted Joining of Polymer–Metal Composites

**DOI:** 10.3390/ma13214875

**Published:** 2020-10-30

**Authors:** Klaus Schricker, Mohammad Alhomsi, Jean Pierre Bergmann

**Affiliations:** Production Technology Group, Department of Mechanical Engineering, Technische Universität Ilmenau, Gustav-Kirchhoff-Platz 2, 98693 Ilmenau, Germany; info.fertigungstechnik@tu-ilmenau.de (M.A.); jeanpierre.bergmann@tu-ilmenau.de (J.P.B.)

**Keywords:** laser, joining, welding, polymer, metal, thermal efficiency, dimensionless numbers, hybrid, composite, heat transfer

## Abstract

Heat conduction joining is mainly used in laser-based joining of metals with polymers but results in a large amount of dissipated heat. The consideration of thermal efficiency allows the determination of power actually used for creating the joint, which is highly relevant for technical and economic reasons, e.g., for calculating the carbon footprint. In order to describe the thermal efficiency universally, process parameters (focal diameter, joining speed, energy per unit length), metallic materials (AA 6082, AISI 304), geometric parameters (overlap width, material thickness) and various polymers (polypropylene, polyamide 6, polyamide 6.6) were examined experimentally. The discussion of the results is supplemented by numerical simulations of the temperature field. For a general description of the physical relationships, some dimensionless numbers based on the Buckingham π theorem were developed, applied to the experimental data. One of these numbers shows similarity to the Fourier number and provides further information on thermal efficiency and its general understanding in the context of polymer–metal joints, enabling the physical background dissipated to stored heat.

## 1. Introduction

Polymer–metal hybrid composites are gaining importance in several fields of application and the motivation for this is manifold. On the one hand, the lightweight potential of such composites can be exploited in novel constructions, e.g., in the automotive or aviation industries. On the other hand, functional integration and cost reduction can be achieved for large series products, e.g., in household appliance technology or the electronics industry.

There are different joining approaches for different material groups to realize polymer–metal composites. In addition to mechanical joining processes [1] and adhesive bonding [2], thermal direct joining has great potential for application in thermoplastic–metal hybrid joints [3]. By saving on auxiliary joining elements such as screws or rivets and on filler materials such as adhesives, a direct bond between polymer and metal can be created [4]. Numerous energy sources can be used in thermal joining [4,5,6]. For industrial applications, however, laser beams have great advantages over alternative processes due to the non-contact energy input and the high degree of flexibility with regard to the components to be manufactured [7].

**Figure 1:**
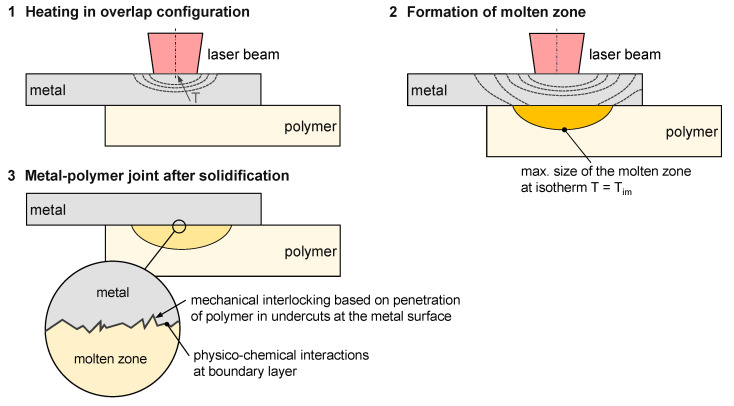
Process steps of laser-based metal–polymer joining.

In laser-based heat conduction joining, polymer and metal are in contact in overlap configuration (Figure 1). The laser beam is focused on the metal surface, and the sheet heats up and the polymer starts melting at the interface due to the heat conduction between the two joining partners. The molten material can now wet the metal surface and penetrate surface structures already present. The geometry of the molten zone in the polymer follows the temperature distribution within the joining zone (schematically drawn as isotherms) and is therefore present at temperatures above the beginning of the melting interval T_im_ [8]. With cooling and solidification, a solid composite of polymer and metal is formed by mechanical interlocking at the surface (form fit) [9] and the physico-chemical interactions (e.g., bonds between aluminium oxide layer and polyamide 6.6) [10]. Due to the different applications, numerous materials are the focus of interest, e.g., high-alloy steels [11] or aluminium alloys [12] as metallic joining partners as well as polyamides [13] or polypropylene [14] as thermoplastic joining partners. In contrast to laser transmission joining, the maximum temperatures in the polymer are reduced and joining of polymers with a high proportion of aggregates and reinforcing materials, e.g., talcum or glass fibres, is possible.

Literature on the state of the art includes different studies of the temperature distribution based on temperature measurements [11] and numerical simulation [15,16,17]. The complex heat conduction conditions in joining technology preclude the use of analytical solutions to describe the temperature field sufficiently. However, the application of dimensionless numbers for the description of heat and mass transfer problems and the comparison of different process parameters is known from laser beam welding [18] and additive manufacturing [19]. These dimensionless numbers can be developed by the Buckingham π theorem [20,21]; familiar parameters such as the Fourier number or the Péclet number are typically used.

The Fourier number (Fo, Equation (1)) expresses the relation between the heat dissipation rate and the heat storage rate [22], where α is the thermal diffusivity (Equation (2)), τ the characteristic time and L the characteristic length. The thermal diffusivity α is the thermal conductivity divided by density ρ and specific heat capacity c_p_. An increasing Fo indicates a higher cooling rate, a higher temperature gradient and less heat accumulation [23]: it affects such characteristics as the melt pool shape in laser welding [24]. The characteristic length L is chosen according to the specific situation, e.g., the melt pool length [23] or focal diameter [25].

(1) $$\mathrm{Fo}=\alpha \cdot \tau \cdot L^{-2}$$

(2) $$\alpha =\lambda \cdot \rho^{-1}\cdot c_{p}^{-1}$$

In contrast, the Péclet number (Pe, Equation (3)) represents the ratio of heat transfer by convection to conduction [19], where L is the characteristic length, α is the thermal diffusivity of the material and v is the characteristic velocity. In laser welding, the weld seam width [26], the focal diameter [27] and the molten bath diameter [28] are among the parameters that can be define the characteristic length L. The Pe is used, among other things, to estimate the length of the molten bath in relation to its width [27] or to obtain an indication of thermal efficiency [29,30].

(3) $$\mathrm{Pe}=L\cdot v\cdot \alpha^{-1}$$

The thermal efficiency is of particular interest. On the one hand, it allows the determination of the proportion of energy input used for actual material processing [31]. On the other hand, the power dissipation can be estimated, which in turn can cause adverse effects, e.g., thermal distortion during welding [22,26]. According to [29], thermal efficiency describes the ratio of process power P_p_ to absorbed laser beam power P_a_ (Equation (4)). The process power P_p_ is used to form the molten zone with the cross-section A_p_ at the joining speed v. Based on [29] and [31] and under the assumptions (a) that the volume of the molten zone is described by the temperature at the beginning of the melting interval T_im_ and (b) that there is no decomposition of the thermoplastic in the joining process, the process power P_p_ follows the representation in Equation (5). The material properties density ρ and specific heat capacity c_p_ are assumed to be constant as mean values. The temperature difference between T_im_ and T_0_ stands for the temperature interval which must be exceeded by the power input of the process to form the molten zone. Because of the solid–liquid phase transformation in this zone, the melting enthalpy ∆H_m_ is taken into account. P_p_ thus stands for the power required to create the molten zone in the polymer in turn creates the joint with the metal. It should be noted that this does not take into account overheating of the polymer or any further thermal effects. The equation thus represents the minimum process power and the minimum thermal efficiency. This has been deliberately chosen to provide a conservative estimation of the CO_2_ balance of the process.

(4) $$\eta_{\mathrm{th}}=P_{p}\cdot P_{a}^{-1}$$

(5) $$P_{p}=A_{p}\cdot v\cdot \rho \cdot \left[c_{p}\cdot \left(T_{\mathrm{im}}-T_{0}\right)+∆H_{m}\right]$$

For laser beam welding, there are numerous studies on the factors influencing thermal efficiency, e.g., welding speed and focal diameter [32]. The thermal efficiency of laser-based polymer–metal joining has so far only been considered to a limited extent. Trials on spot joints [33], which investigated different factors that influence thermal efficiency (e.g., melting interval of the polymer, thermal diffusivity, melting enthalpy) were carried out on the basis of a simplified model material in numerical simulation. The study applied idealized conditions, e.g., by neglecting heat accumulation. The factors assumed to influence thermal efficiency were considered in particular groups, e.g., melting interval and melting enthalpy or material thickness and joining time. For composites of high-alloy steel with the model material, maximum thermal efficiencies of approximately 2 to 3% were achieved without damaging the thermoplastic. The heat conduction losses, in particular via the metallic joining partner, are decisive for the relatively low values due to indirect heating of the polymer part. A general description of the parameters affecting thermal efficiency in laser-based joining of polymers with metals has not yet been given.

In this paper, systematic assessments of the thermal efficiency for laser-assisted metal–polymer joining are carried out. Different geometric arrangements (overlap width, material thickness metal), materials (high-alloy steel, aluminium, the polymers polyamide 6 (PA 6), polyamide 6.6 (PA 6.6) and polypropylene (PP)), focal diameters and energies per unit length are examined. These studies enable the individual factors to be examined individually for their effect on thermal efficiency. On this basis, the data obtained are used to determine general correlations of thermal efficiency by means of newly developed dimensionless numbers and their analogies to the Péclet and Fourier numbers.

## 2. Materials and Methods

**Figure 2:**
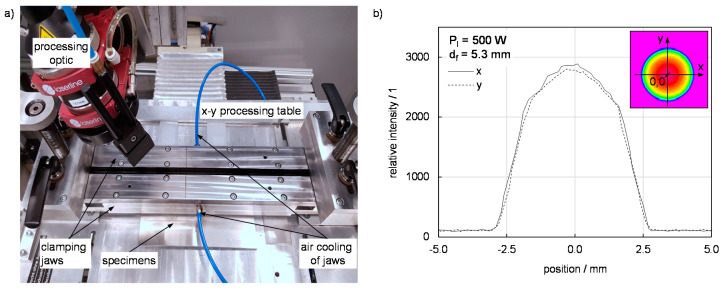
(**a**) Experimental setup and (**b**) schematic view of the joining zone with relevant parameters for thermal efficiency.

The experiments were carried out using a three-axis processing portal (Figure 2a) and a Laserline LDM 1000 diode laser (Laserline, Mülheim-Kärlich, Germany, average wavelength: 980 nm) at a constant beam power P_l_ of 1000 W. Three focal diameters (1.7 mm, 3.5 mm, 5.3 mm) were used and adjusted by different focusing lenses (f = 100 mm, 200 mm, 300 mm). A sample intensity distribution of the laser beam is given in Figure 2b for a focal diameter of 5.3 mm (measured with Primes Beam Monitor at 500 W laser beam power). A clamping device with two air-cooled clamping jaws allowed precise adjustment of the clamping force due to integrated load cells. The size of the specimens was 200 mm in length and 75 mm in width. The overlap width was varied in four steps (14 mm, 24 mm, 36 mm, 75 mm). The main trials were carried out with a complete overlap between both specimens of 75 mm, while further investigations used a smaller overlap width to examine the effect of heat accumulation on thermal efficiency. The joining speed in all cases was adapted to the respective material combination and sheet thickness in steps of at most 0.05 m/min. At low joining speeds the metal is molten, which is considered as exclusion criterion. On the other hand, a minimum width of the melting zone, 3 mm, is specified for increasing joining speeds to ensure a load-bearing connection between the materials. Both conditions are considered due to the application-oriented character of the investigations. Since the focus of the investigation is on thermal efficiency, no surface structuring of the metallic samples is carried out. Metallic as well as polymeric samples are cleaned with isopropyl before joining.

The polymers polyamide 6 (PA 6), polyamide 6.6 (PA 6.6) and polypropylene (PP) are used in several applications, e.g., automotive and domestic appliance industries, and are therefore taken into account. A sheet thickness of 2 mm was chosen. These also have different thermophysical properties, especially the beginning of the melting interval T_im_ (Table 1). This temperature must be exceeded to start the melting of the polymer. Therefore it is considered in the expression of thermal efficiency (Equation (5)). The isotherm of T_im_ is decisive for the maximum expansion of the molten zone [31]. In order to adequately calculate the thermal efficiency, the thermophysical properties of the polymers were calculated as mean values of the interval from 20 °C to T_im_ based on previously published data [34,35,36].

On the metals side, the high alloyed steel AISI 304 (X5CrNi18-10, cold rolled, surface 2B according to EN 10088-2 [37]) was used due to its importance in the household appliance industry. Therefore, material thicknesses of 0.5 mm, 1 mm and 1.5 mm were chosen. The aluminium alloy AA 6082 (heat treatment condition T6 according to EN 515 [38]) was chosen because of its relevance in lightweight applications which is why thicknesses of 1 mm, 1.5 mm and 2 mm were applied. The metallic materials differ significantly in their physical properties, especially with regard to thermal conductivity and thermal diffusivity, which is why a distinct influence on the thermal efficiency can be assumed (Table 1).

**Figure 3:**
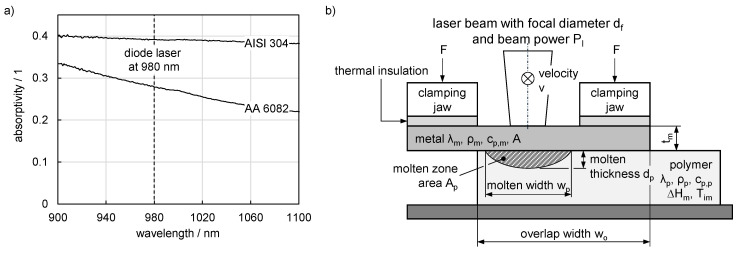
(**a**) Absorptivity and (**b**) schematic view of the joining zone with relevant parameters for thermal efficiency.

Furthermore, the absorptivity A differs significantly between both metals. The reflectance was calculated by reflection measurements in an integrating sphere (spectrophotometer Varian Cary 5000 UV-VIS-NIR, Figure 3a, Agilent, Santa Clara, CA, USA). The transmission is zero and so the absorptivity can be calculated as a function of the wavelength. The absorptivity is assumed to be constant for the wavelength of the diode laser (980 nm) and is approximately 28% for AA 6082 and 39% for AISI 304. Since the laser irradiates the surface at an angle of 15°, the effect of the angle of incidence in the diode laser wavelength is insignificant [39,40]. The changes in absorptivity of steel and aluminium are comparatively small in the experimental temperature interval because the metallic material was not molten during the experiments [30,41]. Therefore, the absorptivity was kept constant at the measured value for both metals. It follows that the absorbed beam power P_a_, which was necessary for the calculation of the thermal efficiency (confer Equation (5)), could be calculated by multiplying the absorptivity A by the laser beam power P_l_ (Equation (6)).

(6) $$P_{a}=A\cdot P_{l}$$

**Figure 4:**
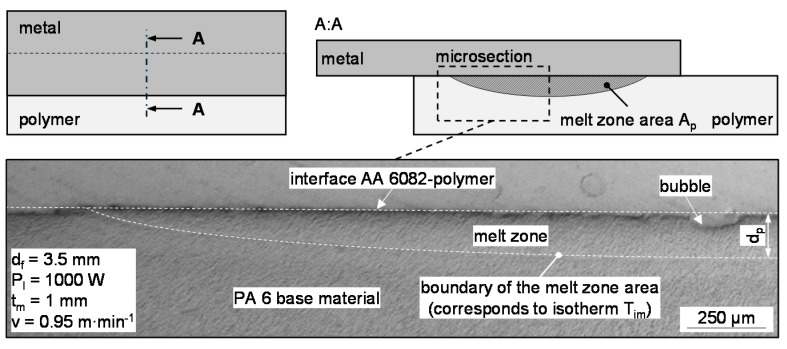
Cross-section of the molten zone in the polymer (metal removed in cross-section).

Figure 3b shows the relevant characteristics of the materials and the process in a cross-sectional view through the clamping device. The thermal insulation was intended to prevent a large loss of heat in the direction of the clamping jaws, which is also typical for clamping devices in this joining process. Regarding properties of importance for the joining partners, the index m indicates the affiliation of the metal and *p* to the polymer material. The area A_p_, width w_p_ and thickness d_p_ of the molten zone are measured in materialographic cross-sections through the middle of the specimens, i.e., after a seam length of 100 mm (Figure 4). The area A_p_ is included in the calculation of thermal efficiency (Equation (5)). As shown in Figure 4, any bubbles that occur are included in the area as they have a minor effect on the resulting volume of the molten zone [34]. These three measured variables are taken into account in the development of dimensionless numbers.

Further variables were also considered in the development of new dimensionless numbers. The energy per unit length E was defined as laser beam power P_l_ divided by joining velocity v (Equation (7)). E is a widely used parameter in joining technology: it establishes an application-oriented relationship between laser beam power and joining speed and enables a direct comparison between the two metals used.

(7) $$E=P_{l}\cdot v^{-1}$$

The interaction time of the laser beam τ_l_ at the surface (Equation (8)) was defined as focal diameter d_f_ divided by joining velocity v. This allowed the introduction of a time-dependent parameter for the dimensionless numbers.

(8) $$\tau_{l}=d_{f}\cdot v^{-1}$$

A sample size of one (*n* = 1) was chosen for the experiments to determine the effect of sheet thickness t_m_, focal diameter d_f_, energy per unit length E, polymer type and overlap width w_o_. This decision was possible because in materialography the process has a very small standard deviation for the evaluation quantities considered; at the same time more steps in the experimental parameters could be considered [33].

The dimensionless numbers were calculated by applying the Buckingham π theorem following [21]. The identified influencing variables (see Figure 3b) are summarized in Table 2 with their SI base units (M: mass, L: length, T: time, θ: temperature) and subdivided into categories for a better overview. From this parameter pool, nine dimensionless numbers were derived as discussed in Section 3.4. The application of the Buckingham π theorem offers the advantage that the characteristic lengths are automatically determined if analogies to known dimensionless numbers like Fourier number are found. These characteristic lengths can deviate from those of previous investigations (see Section 1) due to the indirect joining process, i.e., the laser beam is absorbed at the metal surface and the polymer is heated only by heat conduction.

Due to the wide range of parameters and material properties that were investigated, double-logarithmic graphs are used in order to be able to present the results of the dimensionless numbers in a compact way. When regression curves are given, the coefficient of determination R^2^ is specified.

Numerical simulations of the process were performed based on a thermal model to provide further information about the temperature distribution during joining and to support discussion of the results. In order to reduce the calculation times, consideration of a transient thermal model was omitted. Due to the sample length of 200 mm, it was assumed that a steady state was reached from a particular seam length on. Therefore, a stationary model was assumed to provide sufficient information on the temperature field.

The specimens as well as the clamping device were considered simplified as depicted in Figure 3. Both the basic physics and the initial and boundary conditions were based on the model of [15]. The temperature distribution was calculated from the heat equation assuming an ideal heat transfer between metal and polymer. An ideal heat transfer was also used in [16,42] and showed good agreement with experimental investigations. Further dissipated heat flows were considered by convection and thermal radiation. Density, thermal conductivity and specific heat capacity were considered to be dependent on temperature for both metals and polymers. This allowed the solid-liquid phase transition due to the melting enthalpy to be taken into account. The laser beam absorbed at the metal surface was modelled as a heat flux density of constant intensity over focal diameter. The absorption coefficient of the laser beam power at the metal surface was kept constant at the levels as described above.

## 3. Results and Discussion

### 3.1. Effect of Sheet Thickness, Focal Diameter and Energy per Unit Length on Thermal Efficiency

The following experiments are focused directly on thermal efficiency based on 60 experiments with a sample size of 1. General information on the effect of different material properties on the growth and geometry of the molten zone are provided in [33]. In the first step, the effect of sheet thickness and focal diameter on energy per unit length are discussed. The overlap was kept constant at 75 mm, which meant that the joining partners overlapped completely. The process windows can be easily distinguished by the data series which are connected by a dashed line. The different sheet thicknesses t_m_ are subdivided by colour with the focal diameter d_f_ represented by different symbols (□: d_f_ = 5.3 mm, ◯: d_f_ = 3.5 mm). It should be noted that no process window was reached for a focal diameter of 1.7 mm in these tests. This can be explained using aluminium at 2 mm sheet thickness as an example: at 0.35 m·min^−1^, deep penetration welding occurs after a certain seam length; at 0.40 m·min^−1^, heat conduction welding occurs, and at 0.45 m·min^−1^, an insufficient molten zone width below 3 mm is reached. Therefore, the focal diameter of 1.7 mm is not considered in further investigations.

**Figure 5:**
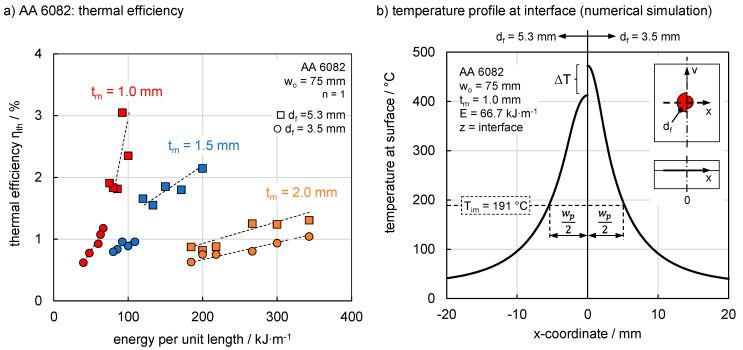
(**a**) The dependence of thermal efficiency on energy per unit length for various sheet thicknesses and focal diameters for the aluminium alloy AA 6082 and (**b**) temperature distribution in the interface for different focal diameters based on numerical simulation.

In general, the thermal efficiency η_th_ increased with rising energy per unit length, but decreased width rising sheet thickness. AA 6082 (Figure 5a) reached a maximum of approximately 3% at a sheet thickness of 1.0 mm. A maximum of approximately 2.1% and 1.4% was obtained at 1.5 mm and 2.0 mm respectively. This behaviour can be explained by the heat that was dissipated in the metal sheet. Increasing thickness led to the input of higher energies per unit length to compensate for the heat loss. For example, the creation of a molten zone with an area of 1.5 mm^2^ required an energy per unit length of 100 kJ·m^−1^ at 1.0 mm while 218 kJ·m^−1^ was necessary at 2.0 mm sheet thickness. A thicker sheet basically led to an increase in molten zone width due to the higher progression of isotherms [33] and an increase in the width of the process window. It should be noted that thermal distortion at 1.0 mm induced deviations in the results and limited the process window due to a change in heat conduction between the materials. On the one hand, higher thermal efficiencies were reached. Constant steps of 0.05 m·min^−1^ in joining velocity led to different maximum thermal efficiencies. Smaller gradations of the joining speeds could provide further information about the maximum thermal efficiency achievable in practice, but within the scope of the observations the basic relationships are recognised.

It is noticeable that the process windows also shifted depending on the focal diameter. While considerably lower energies per unit length were applied with a smaller focal diameter for sheet thicknesses of 1.0 and 1.5 mm, comparable energies per unit length for both spot sizes could be used at 2.0 mm. The reason for this behaviour can be found in the resulting temperature field. A smaller focal diameter led to higher maximum temperatures at the surface and therefore the melting of the metal was reached at lower energies per unit length for 3.5 mm compared to 5.3 mm. The behaviour continues to the interface, where lower maximum temperatures were reached at larger spots. This is shown for example as ∆T in Figure 5b for a constant energy per unit length of 66.7 kJ·m^−1^. On the other hand, the effect of heat sink increased with sheet thickness. This led to a reduction of the maximum temperatures and a comparable process window for both focal diameters was achieved at 2 mm thickness. However the temperature distribution for a larger focal diameter led to a slightly wider temperature distribution of 7% for T_im_ at the interface between the materials where the molten zone formed. This is also illustrated for both focal diameters in Figure 5b by half of the molten zone width (w_p_/2). This resulted in an overall larger molten zone and explains the slightly higher thermal efficiency in case of the larger focal diameter.

**Figure 6:**
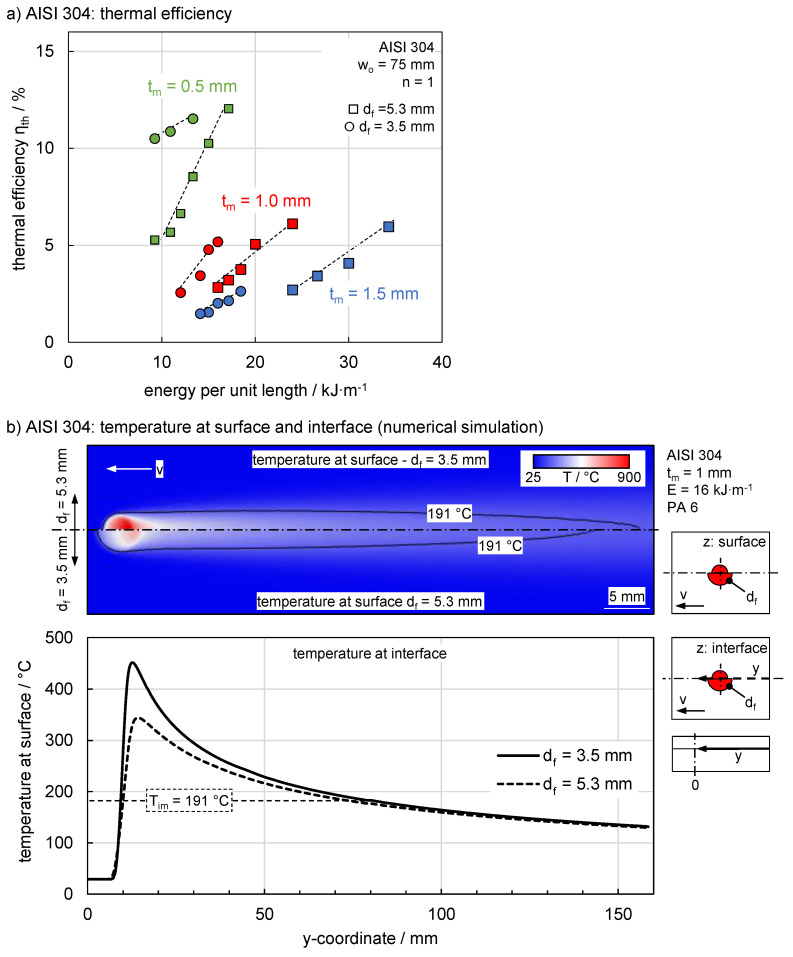
(**a**) Thermal efficiency depending on energy per unit length under varying sheet thickness and focal diameter for the high alloyed steel AISI 304 and (**b**) temperature distribution at the surface and in the interface for different focal diameters based on numercial simulations.

A comparison with the steel illustrates the effect of the metallic material on thermal efficiency (Figure 6a). Although AISI 304 has an 39% higher absorptivity compared to AA 6082, just 10% of the energy per unit length compared to aluminium was required. At the same time, the thermal efficiency increases significantly and reaches a maximum of 12%. The changes in the thermophysical properties and in particular the major reduction of 92% in thermal conductivity compared to AA 6082 are decisive for this behaviour. Thus, the dissipated heat is reduced significantly, and a higher amount of power is used for molten zone formation.

The process windows of lower sheet thicknesses shifted to lower energies per unit length as with AA 6082 (Figure 6a). However, the influence of the focal diameter is different compared to AA 6082, i.e., with a smaller focal diameter, comparable thermal efficiencies were achieved at lower energies per unit length. The reason for this behaviour is based on the different characteristics of the temperature field compared to AA6082. Figure 6b shows an overhead view of the simulated temperature field on the metal surface. The temperature field is represented by the isotherm T_im_ at 191 °C as the polymer begins to melt above this temperature. The width of the temperature field is comparable for both spot sizes despite a significantly higher maximum temperature of the smaller focal diameter due to the low thermal diffusivity and thermal conductivity of AISI 304. The increased maximum temperature led to an extended length of the characteristic isotherm in the direction of movement. From this effect, the temperature distribution of the interface in this direction was considered. The low thermal conductivity led to a significant reduction of the maximum temperature compared to the surface, while it even exceeded the decomposition temperature of 400 °C for a focal diameter of 3.5 mm. On the other hand, it can be seen that the characteristic temperature T_im_ was exceeded for a longer time and at higher temperatures. This led to a larger melt pool depth in which the melt pool width remained constant for both focal diameters. These mechanisms explain the increased efficiency at smaller focal diameter for AISI 304 and are consistent with the experimental results. It should be noted that the increase of the melting zone depth was of minor importance for the application, since the connected area was not increased at this point; this is however decisive for composite production.

### 3.2. Effect of Polymer on Thermal Efficiency

**Figure 7:**
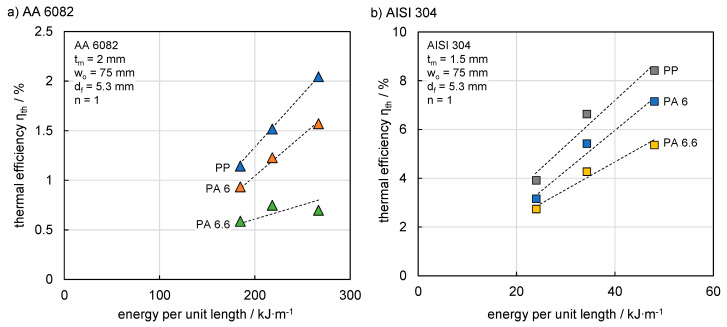
(**a**) Effect of polymer material on thermal efficiency for AA 6082 and (**b**) AISI 304.

The effect of different polymers on thermal efficiency was investigated on a sample basis of 18 experiments with a sample size of 1. Figure 7a shows the thermal efficiency for AA 6082 joints with PP, PA 6 and PA 6.6. It can be seen that a rising energy per unit length led to an increase in thermal efficiency. At the same time, the thermal efficiency was affected by the thermophysical properties of the polymer, especially the melting interval. The beginning of the melting interval T_im_ is the temperature which must be exceeded for the formation of the molten zone. Therefore, thermal efficiency is higher for lower T_im_. PP correspondingly showed the lowest value (T_im_ = 126 K), followed by PA 6 (T_im_ = 191 K) and PA 6.6 (T_im_ = 235 K). The relationship applied equally to AISI 304 (Figure 7b), although higher thermal efficiencies were achieved due to a reduction in heat dissipation in the high alloyed steel. It follows that higher joining speeds can be addressed by joining polymers with a low T_im_ and metals with a low thermal conductivity, producing a comparable molten zone area.

The thermal diffusivity of the polymers also differed (a_PP_ = 1.15 × 10^−7^ m^2^∙s^−1^, a_PA 6_ = 1.39 × 10^−7^ m^2^∙s^−1^ and a_PA 6.6_ = 1.23 × 10^−7^ m^2^∙s^−1^), however, no systematic pattern to the results could be discerned. The same applied to the melting enthalpy; however, this parameter is of minor importance for the size of the molten zone according to [33]. Further information is provided by the consideration of the calculated dimensionless numbers in Section 3.4.

### 3.3. Effect of Overlap Width on Thermal Efficiency

The overlap width along with the specimen length determines the contact surface of the joining partners. The test series consists of 24 experiments with a sample size of 1. A reduced overlap joint can generate heat accumulation at the sheet edge. The laser beam was always positioned in the middle of the overlap. The heat dissipated from the joining zone thus reached the metal edge, which was in contact with the polymer, before it reached the free edge. Both factors affect the temperature field and the dissipated heat. The overlap width was therefore varied for each material thickness. The energy per unit length for the respective metallic joining partner was kept constant for better comparability and to isolate the influence of the overlap width. This resulted in a relatively low thermal efficiency for thicker sheets at an overlap width of 75 mm. However, the overall process remained comparable and melting of the metal sheet for decreased overlap widths was avoided.

**Figure 8:**
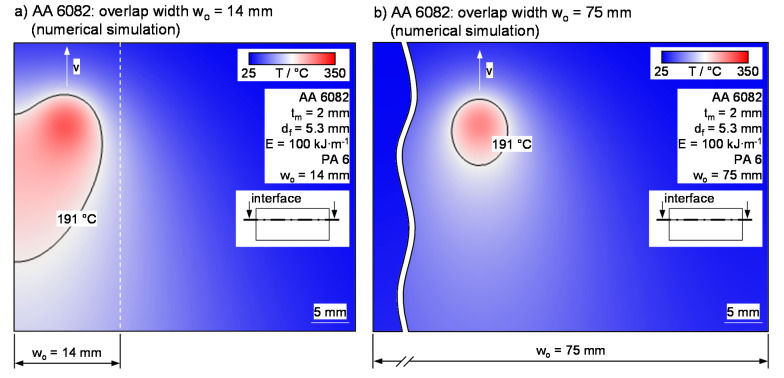
(**a**) Temperature distribution in the interface for an AA 6082–PA 6 joint width overlap widths of 14 and (**b**) 75 mm based on numerical simulations.

Figure 8 illustrates this based on the temperature field at the interface for an AA 6082-PA 6 joint with an overlap of 14 and 75 mm. At constant joining parameters, the heat accumulation at the sheet edge with reduced overlap is clearly observed, compared to the symmetrically formed temperature distribution of the large overlap. At the same time, the propagation of the molten area at the interface, characterized by the 191 °C isotherm, increases significantly. Therefore, a great impact on the thermal efficiency is expected.

**Figure 9:**
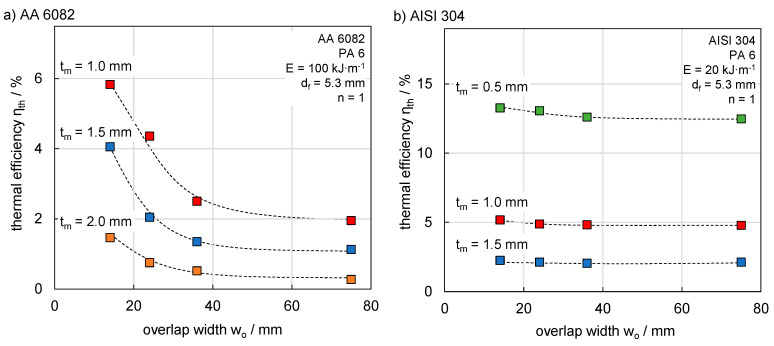
(**a**) The dependence of thermal efficiency on overlap width with constant energy per unit length for AA 6082 and (**b**) AISI 304.

Figure 9a provides the results for AA 6082. The high thermal conductivity of aluminium led to a strong effect of overlap width on thermal efficiency. For 1.0 mm, the thermal efficiency increased from 2% up to 5.8%. The characteristic curve progression was comparable for all the sheet thicknesses investigated, i.e., the thermal efficiency increased with smaller overlap width due to the heat accumulation from the reduced heat loss. In comparison, AISI 304 showed a different behaviour (Figure 9b). A decrease of the overlap width showed just a slight effect on thermal efficiency because of the significantly lower thermal conductivity. For the same reason, higher maximum values of η_th_ were reached for the high alloyed steel. The thermal efficiency was nearly constant for changing overlap. Even for a sheet thickness of 0.5 mm, the increase in thermal efficiency was 0.8% between 14 mm and 75 mm overlap width.

### 3.4. General Description of Thermal Efficiency by Dimensionless Numbers

**Figure 10:**
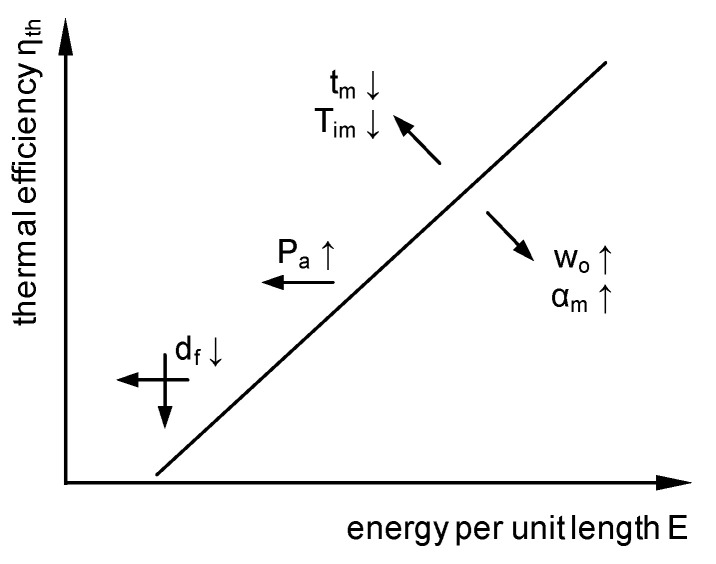
Qualitative illustration of the influencing parameters on thermal efficiency.

Based on the experimental results, the parameters investigated were qualitatively pooled in Figure 10 regarding their effect on thermal efficiency. In general, a minimum energy per unit length was required before the polymer melted. The thermal efficiency then increased approximately linearly in the field under consideration. An increase in the absorbed laser beam power P_a_ thus shifted the curve to the left. Both a reduction of the metal sheet thickness t_m_ and a lowering of the melting interval of the polymer, characterized by the beginning of the melting interval T_im_, contributed to an increase of thermal efficiency. In contrast, an increase in the overlap width w_o_ led to a reduction in thermal efficiency; this effect was closely related to the thermal diffusivity of the metal material as discussed in Section 3.3. In contrast, an increase in thermal diffusivity led to a reduction in thermal efficiency due to the enhanced heat conduction loss. The influence of the focal diameter was ambivalent for different thermal diffusivities due to the temperature field formed and could lead to either an increase or a decrease of thermal efficiency (see Section 3.1).

Figure 10 does not show quantification across different materials, parameters and ambivalent effects. To provide a quantified and generally valid description of the thermal efficiency in laser-based joining of polymers and metals, dimensionless numbers were calculated by means of Buckingham π theorem (see Section 2). The Buckingham π theorem provides a method for calculating dimensionless numbers based on given variables with known dimensions, even if the form of the equation is unknown. The theorem states, among other things, that any physical law can be expressed by dimensionless indices or their combination, on the condition that the physical problem is modelled considering the correct parameters. This provides a simple way of doing dimensional analysis, but does not provide a direct description of the physical relationship or of the importance of the dimensionless numbers. In order to determine specific relationships, all dimensionless numbers were calculated using the parameters of each experiment and compared to the thermal efficiency. Therefore, the dimensionless numbers were applied to the complete data set consisting of all test series from Section 3.1, Section 3.2 and Section 3.3, in order to check the general validity regarding all different parameters considered. It should be noted that the aggregated results also contain data sets that have not previously been presented and which were created by increasing the sample size for individual experiments. This results in a total of 276 experiments, which were accordingly materialographically evaluated. The aim was to provide a broader database for the validation of the dimensionless numbers.

**Figure 11:**
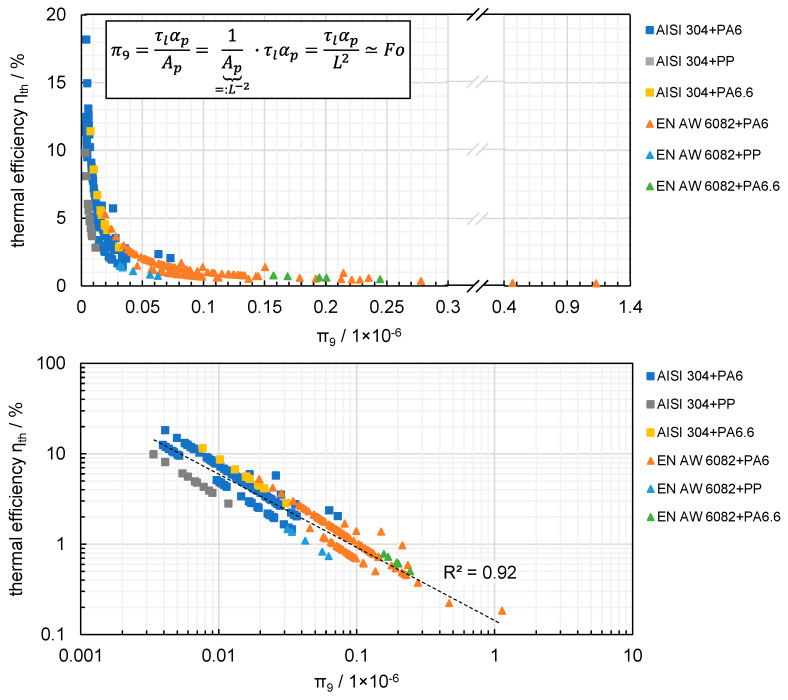
The dependence of thermal efficiency on π_9_ in linear (**top**) and logarithmic (**bottom**) representations.

The derived numbers are given in Table 3. The evaluation showed that π_1_ to π_8_ did not provide a generally valid relationship with thermal efficiency. In contrast, π_9_ provided interesting results regarding thermal efficiency as shown in Figure 11. To show the results most clearly, the upper diagram has linear axes while the lower has logarithmic axes, and different colours highlight various material combinations. The dimensionless number π_9_ allows a correlation of the metals used, aluminium and steel, even though they have different material properties. Due to the higher thermal efficiencies, steel is mainly located on the left of the diagram, with aluminium on the right. Nevertheless, there is a considerable transition area in the range of approximately 0.03–0.06 × 10^−6^ of the dimensionless number π_9_. In the logarithmic representation, it is evident that the correlation is equally valid for different material combinations. However the coefficient of determination of 0.92 should not hide the fact that the vast majority of experiments were carried out on two combinations of materials (AISI 304 with PA 6, EN AW 6082 with PA 6), which favoured the high value. Nevertheless, all test series–overall and in detail–followed an exponential function.

A more detailed examination of π_9_ reveals a similarity to the Fourier number (Fo, Equation (1)) which describes the ratio of dissipated to stored heat (see Section 1) as shown in Figure 11 (top). Compared to the characteristic length L, which enters the Fo quadratically, the Buckingham π theorem led to the molten zone area A_p_ taken into account. This area was melted by the energy input in the indirect joining process, i.e., the heat was conducted through the metal into the polymer, which is why it can also be seen as a characteristic quantity of the process. The calculation of the thermal efficiency is also based on it (Equations (4) and (5)). For small values of π_9_, the ratio of stored to dissipated heat results in a higher thermal efficiency; for larger values of π_9_, the opposite applies. In addition to the interaction time of the laser beam, the thermal diffusivity of the polymer was also taken into account. It can be assumed that propagation of the temperature field was also reflected in the molten zone area, since it directly causes its width [33]. The dimensionless number thus provides a description of thermal efficiency across different material combinations, material properties, joining parameters and geometric variables. 

Alternative trials with other model approaches, e.g., considering the joining velocity, could also establish dimensionless numbers similar to the Péclet number (Pe, Equation (2)). However, a general validity for both metallic materials was not reached. 

It should be noted that dimensionless numbers can combine various factors and even boundary conditions. This is noticeable in that the trials on overlap width are integrated into the characteristic number π_9_ without major deviations. An extension of the thermal analysis to thermal-mechanical questions, e.g., the influence of boundary conditions on hot cracking as shown in [43], is conceivable in principle. However, the modelling of such questions is more difficult due to the fact that decisive factors on the mechanical processes, e.g., the material history, cannot be determined directly.

## 4. Conclusions

In this paper, the thermal efficiency of laser-based joining of polymers with metals was investigated. Based on individual test series on the influence of sheet thickness, focal diameter and energy per unit length, different polymers as well as various overlap conditions were considered. The partially opposing effects on thermal efficiency were clarified by numerical simulation of the temperature distribution. The methodology that was developed allowed the comparison of strongly differing material properties, e.g., the absorbed laser beam power for the comparison of aluminium and steel materials. Factors that had a decisive effect on the thermal efficiency were identified and a qualitative model was developed.

In addition, a quantitative description of thermal efficiency was achieved by applying the Buckingham π theorem. The development of a dimensionless number enabled all test series to be incorporated. This characteristic number shows similarity to the Fourier number, enabling the physical background of dissipated to stored heat. This model enables the thermal efficiency of the process to be described in general, but also allows measures for increasing the thermal efficiency and to be derived and used for the evaluation of the joining process, e.g., for calculation of the carbon footprint.

## Figures and Tables

**Table 1:** Material properties [34,35,36].

Material Property	Symbol	Unit	AISI 304	AA 6082	PA 6	PA 6.6	PP
thermal conductivity ^1^	λ	W∙m^−1^∙K^−1^	15	185	0.35	0.31	0.24
specific heat capacity ^1^	c_p_	J∙kg^−1^∙K^−1^	470	896	1700	1670	2090
density ^1^	ρ	kg∙m^−3^	7900	2700	1130	1140	910
beginning of melting interval ^2^	T_im_	°C	1400	585	191	235	126
melting enthalpy ^2^	∆H_m_	kJ∙kg^−1^	- ^3^	- ^3^	50	47	87
absorptivity ^3,4^	A	1	0.39	0.28	- ^3^	- ^3^	- ^3^

^1^ at room temperature, ^2^ determined by DSC analysis at 10 K∙min^−1^ heat rate according to [34], ^3^ not relevant in the context of the study, ^4^ at wavelength of 980 nm.

**Table 2:** Relevant parameters for dimensionless numbers.

Category	Variable	Symbol	Unit	SI Base Unit ^1^
process parameters	absorbed laser beam power	P_a_	W	M∙L^2^∙T^−3^
	interaction time	τ_l_	s	T
joint configuration	overlap width	w_o_	m	L
	thickness of metal sheet	t_m_	m	L
material properties of the metal	thermal conductivity	λ_m_	W∙m^−1^∙K^−1^	M∙L∙T^−3^∙θ^−1^
	specific heat capacity	c_p,m_	J∙kg^−1^∙K^−1^	L^2^∙T^−2^∙θ^−1^
	density	ρ_m_	kg∙m^−3^	M∙L^−3^
material properties of the polymer	thermal diffusivity	α_p_	m^2^∙s^−1^	L^2^∙T^−1^
	beginning of melting interval	T_im_	°C	θ
	melting enthalpy	∆H_m_	kJ∙kg^−1^	L^2^∙T^−2^
resulting molten zone	molten zone area	A_p_	m^2^	L^2^
	molten zone width	w_p_	m	L
	molten zone thickness	d_p_	m	L

^1^ SI base units: M–mass, L–length, T–time, θ–temperature.

**Table 3:** Calculated dimensionless numbers π_i_.

Number π_i_	Formula
π_1_	t_m_∙w_o_^−1^
π_2_	w_o_^5^∙ρ_m_∙P_a_∙τ_l_^−3^
π_3_	P_a_∙τ_l_^2^∙c_p,m_∙w_o_^−3^∙λ_m_^−1^
π_4_	w_p_∙w_o_^−1^
π_5_	τ_l_^2^∙∆H∙w_o_^−2^
π_6_	w_o_∙λ_m_∙T_im_∙P_a_^−1^
π_7_	w_o_^2^∙A_p_^−1^
π_8_	d_p_∙w_o_^−1^
π_9_	τ_l_∙α_p_∙A_p_^−1^
